# Comparison of ESWL and Ureteroscopic Holmium Laser lithotripsy in Management of Ureteral Stones

**DOI:** 10.1371/journal.pone.0087634

**Published:** 2014-02-03

**Authors:** Yon Cui, Wenzhou Cao, Hua Shen, Jianjun Xie, Tamara S. Adams, Yuanyuan Zhang, Qiang Shao

**Affiliations:** 1 Department of Urology, Suzhou Municipal Hospital, Suzhou, China; 2 Center for Cancer Genomics, Wake Forest University School of Medicine, Winston-Salem, North Carolina, United States of America; 3 Wake Forest Institute for Regenerative Medicine, Wake Forest University Health Sciences, Winston-Salem, North Carolina, United States of America; Glasgow University, United Kingdom

## Abstract

**Background:**

There are many options for urologists to treat ureteral stones that range from 8 mm to 15 mm, including ESWL and ureteroscopic holmium laser lithotripsy. While both ESWL and ureteroscopy are effective and minimally invasive procedures, there is still controversy over which one is more suitable for ureteral stones.

**Objective:**

To perform a retrospective study to compare the efficiency, safety and complications using ESWL *vs.* ureteroscopic holmium laser lithotripsy in management of ureteral stones.

**Methods:**

Between October 2010 and October 2012, 160 patients who underwent ESWL or ureteroscopic holmium laser lithotripsy at Suzhou municipal hospital for a single radiopaque ureteral stone (the size 8–15 mm) were evaluated. All patients were followed up with ultrasonography for six months. Stone clearance rate, costs and complications were compared.

**Results:**

Similarity in stone clearance rate and treatment time between the two procedures; overall procedural time, analgesia requirement and total cost were significantly different. Renal colic and gross hematuria were more frequent with ESWL while voiding symptoms were more frequent with ureteroscopy. Both procedures used for ureteral stones ranging from 8 to 15 mm were safe and minimally invasive.

**Conclusion:**

ESWL remains first line therapy for proximal ureteral stones while ureteroscopic holmium laser lithotripsy costs more. To determining which one is preferable depends on not only stone characteristics but also patient acceptance and cost-effectiveness ratio.

## Introduction

Urolithiasis has plagued human beings for thousands of years. Advances in medicine have enabled us to better treat urolithiasis with few complications. Most urinary stones that pass through the renal calyces to the renal pelvis and subsequently to the ureter cause serious symptoms. The most common symptoms of ureteral stones are pain, hydronephrosis and hematuria. There are many options for urologists to treat ureteral stones that range from 8 mm to 15 mm (Table 1), including ESWL and ureteroscopic holmium laser lithotripsy [1], [2], [3]. While ESWL and ureteroscopy are effective and minimally invasive procedures, there is still controversy over which one is more suitable for ureteral stones. Different studies have reported variable outcomes of ESWL and ureteroscopy, despite the fact that both treatments use advanced instruments, offer few complications and high satisfaction among urologists [4]–[8]. We conducted the current study to compare objective outcomes of patients with ureteral stones treated with ESWL or ureteroscopy.

**Table 1:** Management of Ureteral Calculi: Guidelines on Urolithiasis of EAU, AUA and CUA.

	Proximal ureter	Mid-ureter	Distal ureter	
	(All size)	(All size)	(All size)	
EAU options	ESWL, URS, PNL, Lapa and open surgery	ESWL, URS, PNL	URS, ESWL	
	**Proximal ureter**		**Distal ureter**	
	**<1 cm**	**>1 cm**	**<1 cm**	**>1 m**
AUA options	Observation,ESWL	ESWL,URS,PNL,Laparoscopic and open surgery	Observation, URS, ESWL	URS, ESWL
	**Proximal ureter**		**Distal ureter**	
	**<0.8 cm**	**>0.8 cm**	**<0.8 cm**	**>0.8 cm**
CUA options	Observation, ESWL	ESWL, URS, PNL, Laparoscopic and open surgery	Observation, URS	URS

Note: EAU- European Association of Urology; AUA-American Urological Association; CUA-Chinese Urological Association; URS-Ureteroscopy; PNL- Percutaneous nephrolithotomy; Lapa-laparoscopy.

## Materials and Methods

### Study Population

Patients that underwent ESWL (n = 80) and ureteroscopy (n = 80) between October 2010 and October 2012 at Suzhou Municipal Hospital for a single radiopaque ureteral stone ranging from 8 mm to 15 mm were included in the study (Table 2). Patients with bilateral or multiple stones, radiolucent stones, ureteral stricture, acute urinary tract infection, repeated treatment and distal ureteral stones were excluded. Patients were provided with an explanation of the advantages, drawbacks, and complications associated with each procedure prior to voluntarily selecting a treatment option. The study protocol was approved by the Institutional Review Board of Suzhou Municipal Hospital and written informed consent was obtained before treatment.

**Table 2:** Characteristics of the patients on both groups.

	ESWL	Ureteroscopy	*P* value
Age(year),	40.6±9.8	41.5±10.5	0.69
Sex, male/female	49/31	56/24	0.478
Stone site			0.302
Right	38(47.5%)	34(42.5%)	
Left	42(52.5%)	46(57.5%)	
Stone location			0.446
Upper ureter	56(70.0%)	62(77.5%)	
Mid-ureter	24(30.0%)	18(22.5%)	
Stone diameter(mm)	9.8±3.5	10.2±4.3	0.65

### Study Design

**Figure 1:**
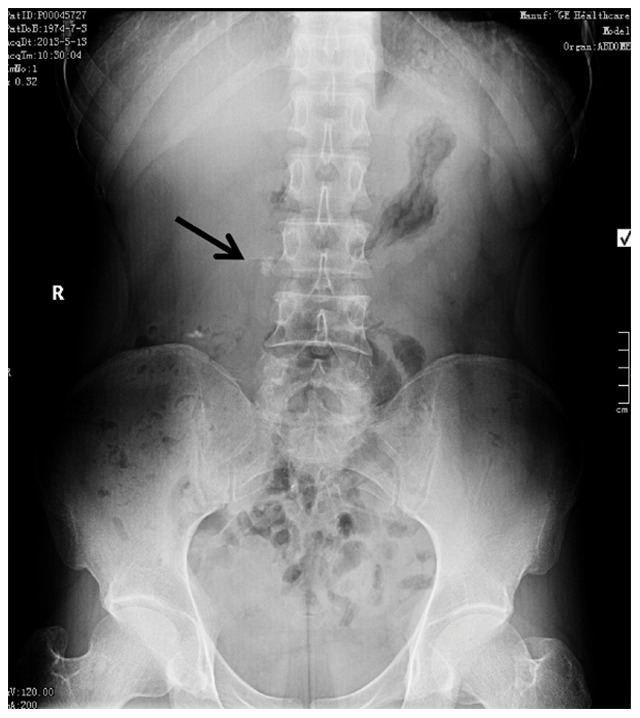
Rightureteral calculus (arrow) before ESWL.

**Figure 2:**
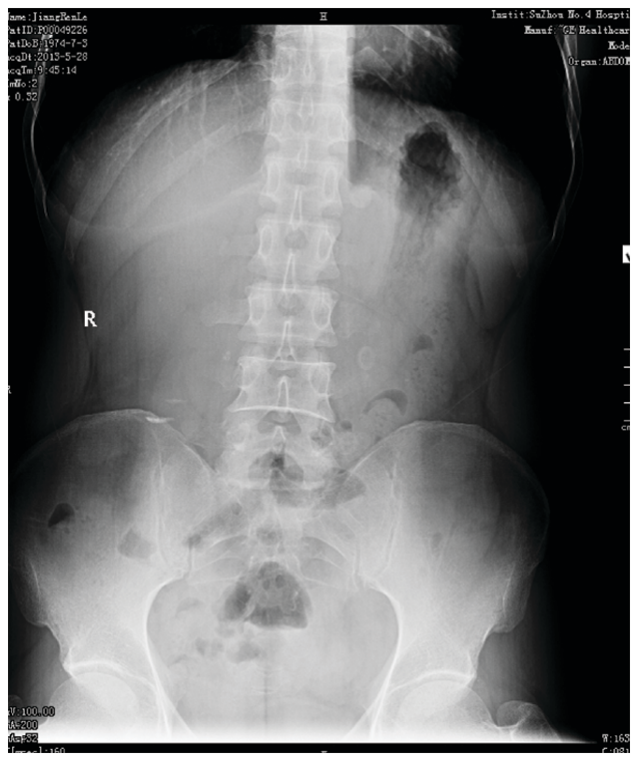
Calculusafter ESWL.

Ureteral stones were diagnosed by Kidney-Ureter-Bladder (KUB) X-Ray film (Figure 1) and ultrasonography then the longest diameter of each stone was calculated before treatment. Patients that underwent ESWL were evaluated by urine analysis, serum creatinine level and coagulation profile. In addition, ESWL was performed using the third-generation Dornier lithotripter (Dornier, Germany) as an outpatient procedure. Voltage was set at 10–12****Kv and shockwave was set at 3,000–3,500 shocks for the first treatment. Patients were discharged after 2 hours surveillance with routine antibiotics and evaluated one week later by KUB to assess stone passage (Figure 2). ESWL was repeated if residual stones were observed at follow-up. Following the guidelines of the Chinese Urology Association (CUA), if a stone remained after three sessions of ESWL, we performed ureteroscopy in order to avoid ureterostenosis [3].

**Figure 3:**
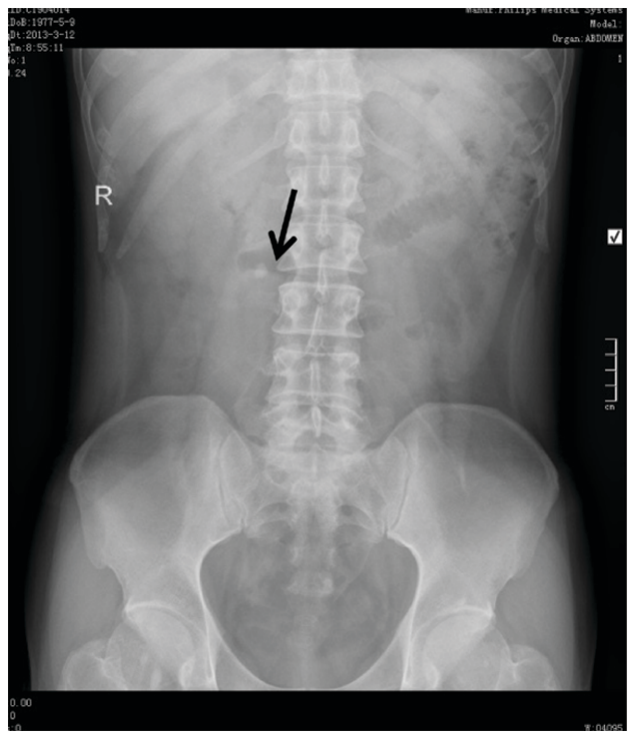
Right ureteral calculus (arrow) before ureteroscopy.

**Figure 4:**
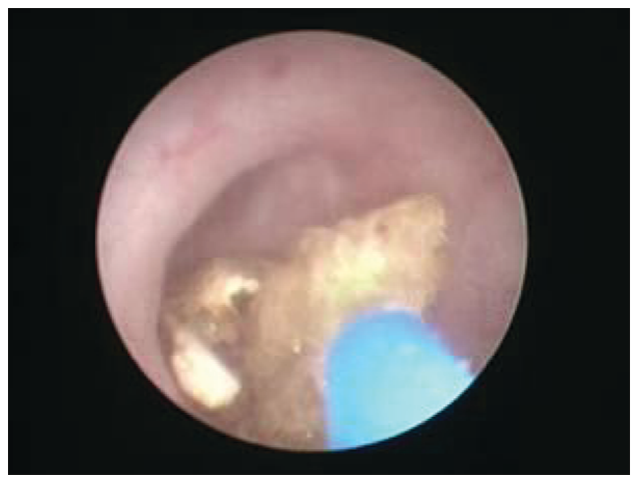
Ureteroscopic holmium laser lithotripsy.

**Figure 5:**
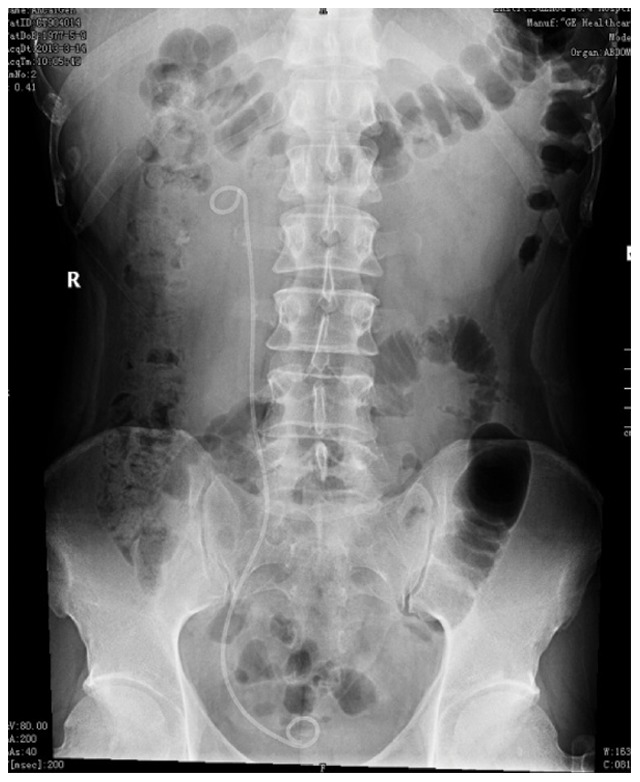
Calculus-freeafter ureteroscopy.

Patients that underwent ureteroscopy were evaluated by urine analysis, serum creatinine level, coagulation profile, EEG, chest radiograph and KUB X-Ray film (Figure 3). Ureteroscopy was performed using 8/9.8-Fr, 12° rigid ureteroscope (Richard Wolf, Germany) under general anesthesia. For ureteroscopic lithotripsy, a holmium laser (Dahua Systems, Wuxi, China) was used at a setting of 1.2–1.5 J and 10–15 Hz. After stones were broken into pieces <2 mm one 6.0F double-J was indwelled (Figure 4). Both antibiotics and analgesia were routinely used. All patients were discharged postoperative 1–2 days after catheter removal. Patients were evaluated by KUB one week postoperative (Figure 5) and the stent were removed postoperative 2 weeks.

### Statistical Analysis

Fisher’s exact or chi-square test was used to compare categorical variables between the groups. The Student’s t test was used to compare continuous variables. All statistical analyses were two-sided with p<0.05 defined as statistically significant. We used SAS software, version 9.2 (SAS, Cary, NC), for statistical analyses.

## Results

Both procedures were successfully achieved at the same time no severe complications during treatment occurred (renal haematoma for ESWL and ureteral perforation or ureteral avulsion for ureteroscopy). The detailed procedural indexes of patients are listed in Table 3. Between the two procedures, overall procedural time, analgesia requirement, total cost, gross hematuria and voiding symptom were significantly different while the stone clearance rate and treatment time were similar. In ureteroscopy group there were two cases which stones shifted to the pelvis and couldn’t be reached by rigid ureteroscope; one week later residual stones were removed by ESWL. In ESWL group there were six cases which stones couldn’t be removed completely finally were managed by ureteroscopy. All patients were followed up for 3–6 months and there were no obvious residual stones or ureterostenosis at the end of follow-up.

**Table 3:** Detailed procedural indexes of patients.

	ESWL	ureteroscopy	*P* value
Stone clearane rate	74(92.5%)	78(97.5%)	0.61
Treatment time(min)	40.0±10.0	42.5±11.3	0.29
overall procedural time(h)	3.0±1.0	48.0±8.5	<0.05
Analgesia(tramadol mg)	0	80.5±31.6	<0.05
Cost(US $)	120±25	1180±258	<0.05
Introprocedural Complications			
renal haematoma	0	0	
ureteral perforation (avulsion)	0	0	
Early complications			
renal colic	9(11.25%)	2(2.5%)	<0.05
Gross hematuria	16(20%)	2(2.5%)	<0.05
Voiding symptom	5(6.25%)	27(33.75%)	<0.05
Late complications			
residual calculus	0	0	
Ureterostenosis	0	0	
post-procedure satisfaction	72(90%)	67(83.75)	>0.05

Note: “overall procedural time” for ESWL include the repeated procedures in some cases; “overall procedural time” for ureteroscopy included anesthesia time, operation time and hospitalization time.

## Discussion

Both ESWL and ureteroscopy are minimally invasive treatment options for patients with proximal ureteral stones. The use of ESWL began in the1980s, has stone clearance rate of nearly 90% and has resulted in the fading of open surgical procedures for ureteral stones [9]–[11]. With subsequent development of ESWL instrument it eliminated the limitations and promoted the efficiencies. Ureteroscopy was first described in 1912 [12], but its use was not widely accepted until the late 1970’s, at which time it became a standardized procedure [13]. Along with laser lithotripsy, an impressive 95% or more of the patients treated with were stone free after a single procedure [14], [15]. Most of the comparative studies between ESWL and ureteroscopy are not conclusive and sometimes ambiguous. While some studies are in favor of ESWL [7], others concluded that ureteroscopy is the preferable approach [16]–[18]. We compared several objective outcomes of each procedure method, including stone clearance rate, treatment time, procedural time, complications and costs.

Stone clearance rates in our study were 92.5% for ESWL versus 97.5% for ureteroscopy and not significantly different. These rates were similar to those in previous studies [15], [16]. In ureteroscopy group we broke stones into pieces<2 mm during surgery, moreover evaluation of stone clearance based on KUB imaging one week later it might improve the stone passage; in ESWL group stone clearance rate evaluated according to final outcomes which patients maybe experience more than one session of ESWL. As Table 4 showed that the stone clearance rate of uerteroscopy was significantly different with one session of ESWL. Multiple sessions of ESWL resulted in a similar outcome to ureteroscopy. This could explain why the stone clearance rate of ESWL varied from 80%–100% [4], [7]. Furthermore, the stone clearance rate of ESWL was determined by stone composition and stone hardness. Holmium laser lithotripsy is a reliable method of stone disintegration irrespective of the stone composition and hardness, it is carried out through all types of ureteroscope [19], but stone migration will make operation failure [20], [21]. With the instrument development and operation skill promotion we still cannot avoid ureteroscopic lithotripsy failure. Ureteroscopy with laser lithotripsy has an advantage over one session of ESWL with regard to stone clearance rate and efficiency [22]–[24], but ESWL still is the first line option for proximal ureteral calculi because of fewer cost and easy performance. Moreover, ESWL will be preferred option when ureteroscopy fails.

**Table 4:** Stone clearance rate according to ESWL sessions.

	ESWL1	ESWL2	ESWL3	Ureteroscopy
Stoneclearance rate (%)	77.5	87.5	92.5	97.5
*P* value(*vs*.ureteroscopy)	0.018	0.20	0.61	

ESWL1∶1^st^ session ESWL and so far.

We did not observe a difference in treatment times between the two groups (Table 3). However, there was a significant difference in total procedural time between both groups. For ESWL group it took the shockwave lithotripsy time and two hours’ surveillance then patients went back home until follow up a week later as well as for ureteroscopy group it included operation and hospitalization time which is much more (3.0±1.0 *vs.* 48.0±8.5 hours). Patients underwent ESWL experienced a fast recovery and returned to daily activity within two days while some patients underwent ureteroscopy suffered from voiding symptoms until the ureteral stent was removed postoperative two weeks [25], [26]. The procedural time difference resulted the cost difference (120±25*vs.*1180±258US $). Even if patients underwent three sessions of ESWL, the cost between the two options was still significantly different. Moreover, as a surgical procedure, ureteroscopy require general anesthesia and post-operation analgesia while as an outpatient procedure, ESWL doesn’t require the both. Conclusively, patients underwent ESWL had a higher cost-effectiveness ratio than patients underwent ureteroscopy.

We compared complications between the two procedures. We didn’t found any severe complications such as renal haematoma for ESWL and ureteral perforation or ureteral avulsion for ureteroscopy during both procedures; secondly we found patients underwent ESWL experienced a high percentage of hematuria and renal colic than patients underwent ureteroscopy. Macroscopic bleeding and renal colic for ESWL were caused by stone fragment movements and ureteral mucosa damage [27], [28], as most stone fragments were cleared during operation fewer hematuria and renal colic were observed after ureteroscopy. Most hematuria and renal colic will vanish after stone passage without special management. Finally we found more patients underwent ureteroscopy suffered voiding symptoms than patients underwent ESWL (67.5% *vs.* 7.5%). In our procedure of ureteroscopy each patient indwelled ureteral stent and moved out two weeks later it should be the reason patients underwent ureteroscopy suffered more voiding symptom. According to Joshi et al and Lamb et al [29], [30] reporter, voiding symptoms and other discomforts after receiving a ureteral stents was unavoidable for ureteroscopy procedure. There is evidence that α-blockers alleviate ureteral stent discomfort, but they don’t work completely [31]. Voiding symptoms might be the reason why many patients stated on a questionnaire that they feared of ureteroscopy. At the end of six months follow up there were no residual stones or ureterostenosis in both groups.

A post-procedure questionnaire revealed a similar satisfaction rate that 90% of patients who underwent ESWL were satisfied with the procedure *vs.* 83.75% of patients who underwent ureteroscopy. Most patients who underwent ureteroscopy felt dissatisfied because of voiding symptoms and higher costs of the procedure. To our knowledge, there are no other reports with similar data in a Chinese cohort. This study was performed in one hospital and only included 160 cases so that all conclusions need further multi-center prospective study to validate.

In conclusion, ESWL as an outpatient procedure does not require analgesia or anesthesia; it remains the first line therapy for proximal ureteral stones while ureteroscopic laser lithotripsy as a surgical procedure requires general anesthesia, hospitalization and much more costs. Determining which procedure is preferable depends on stone characteristics, patient acceptance and cost-effectiveness ratio.
